# Intestinal Mature B-Cell Non-Hodgkin Lymphoma Presenting as an Acute Surgical Abdomen in Children: A Case Series

**DOI:** 10.3390/pediatric18050139

**Published:** 2026-10-14

**Authors:** Izabela Kis, Ivana Jukic, Borna Kovacic, Ivancica Bekavac, Tea Grujic-Tomas, Zenon Pogorelic

**Affiliations:** 1 Faculty of Medicine, Josip Juraj Strossmayer University of Osijek, 31000 Osijek, Croatia; 2 Department of Pediatric Surgery, University Hospital Centre Osijek, 31000 Osijek, Croatia; 3 Department of Abdominal Surgery, University Hospital Centre Osijek, 31000 Osijek, Croatia; 4 Department of Surgery, School of Medicine, University of Split, 21000 Split, Croatia; 5 Department of Pediatric Surgery, University Hospital of Split, 21000 Split, Croatia

**Keywords:** children, non-Hodgkin lymphoma, intestinal obstruction, urgent surgery, B-NHL, mechanical bowel obstruction

## Abstract

Intestinal mature B-cell non-Hodgkin lymphoma (B-NHL) is the most common pediatric gastrointestinal lymphoma, but presentation as acute mechanical bowel obstruction remains rare and still challenging. Evidence guiding emergency surgical management in children is limited to small retrospective series and case reports. This case series describes three children with intestinal B-NHL presenting with acute mechanical bowel obstruction, or intussusception. All three patients presented to the emergency department with signs of bowel obstruction and an acute abdomen, and in one patient initial diagnosis suggested appendicitis. Imaging revealed either intussusception with a lead mass or large obstructing abdominal tumors. Surgery within 8–48 h consisted of segmental bowel resection with primary anastomosis in all cases. The final diagnoses were Burkitt lymphoma in two patients and diffuse large B-cell lymphoma (DLBCL) in one. The wider literature confirms that lymphoma-related intussusception and obstructing ileal masses occur predominantly in older children, are frequently misdiagnosed preoperatively, and often require emergency surgery both for diagnosis and for treatment of obstruction. Available literature has examined outcomes after emergency surgery for intestinal lymphoma, but the present case series cannot determine its independent effect on long-term oncologic prognosis. Pediatric intestinal B-NHL can present as acute mechanical bowel obstruction or intussusception suggesting common surgical conditions. Surgeons play a crucial role in early recognition, appropriate extent of resection, and timely transition to oncologic care. Further multicenter studies are needed to optimize surgical decision-making and quantify outcomes in this rare but life-threatening presentation.

## 1. Introduction

Non-Hodgkin lymphoma has the fourth highest cancer incidence in children and accounts for 7% of cancers in the pediatric population. NHL comprises four wide categories: Burkitt lymphoma, diffuse large B-cell lymphoma (DLBCL), lymphoblastic lymphoma, and anaplastic large cell lymphoma (ALCL) [1,2]. Intestinal non-Hodgkin lymphoma in children is a rare but important cause of acute abdominal emergencies, most often involving the ileum or ileocecal region and typically arising from mature B-cell disease [1,2,3,4]. It often becomes clinically apparent only after it produces obstruction, intussusception, abdominal mass effect, or less commonly perforation, which is why surgeons are frequently involved at first presentation. Primary gastrointestinal tumors are uncommon in childhood, yet NHL is the most common GI malignancy in this age group and has a variable, often misleading abdominal presentation [5,6]. In intestinal disease, symptoms commonly begin with recurrent or prolonged abdominal pain, vomiting, distension, altered bowel habits, gastrointestinal bleeding, weight loss, or a palpable mass, but these findings are nonspecific and can delay recognition while the tumor enlarges [1,2,5,6,7]. Obstruction and intussusception are among the most characteristic surgical presentations of pediatric intestinal NHL [1,3,8]. A bowel wall or intraluminal lymphoma can narrow the lumen directly or serve as a pathological lead point, especially in the distal ileum and ileocecal region, producing acute bowel obstruction, recurrent intussusception, ischemia, bleeding, or necrosis [3,5,8,9]. Several diagnostic traps should raise suspicion for lymphoma rather than idiopathic intussusception. Children with lymphoma-related intussusception tend to be older, more often boys, and more likely to have prolonged, intermittent, or recurrent symptoms than children with idiopathic intussusception [5,10,11,12]. They are also more likely to present with intestinal obstruction, ascites, enlarged mesenteric lymph nodes, or a visible intra-abdominal mass on imaging, and enema reduction is less likely to succeed [3,4,10]. Several reports further note that atypical age, recurrent intussusception, prolonged abdominal pain, and a suspected pathological lead point on ultrasound should specifically raise concern for lymphoma rather than idiopathic disease [3,10,11]. The challenge is recognizing lymphoma before the abdomen declares itself surgically. This makes early suspicion especially important in atypical intussusception or unexplained obstruction. Ultrasound is commonly the initial test for pediatric abdominal mass or suspected intussusception, while CT is used to define the lead point, bowel involvement, and disease extent [3,7,13]. PET/CT contributes mainly to staging rather than first diagnosis, and modern pediatric NHL staging systems explicitly incorporate advanced imaging technologies [6,14,15]. Even with ultrasound and computed tomography (CT), preoperative identification of a malignant lead point remains difficult, especially in older children with small-bowel intussusception, so tissue diagnosis remains necessary [6,7]. Definitive diagnosis still depends on histopathology with immunohistochemistry, which is frequently obtained after urgent surgery or biopsy [3,4,5,6,13]. In this setting, the surgeon has both a diagnostic and therapeutic role: surgery is required for complete obstruction, failed reduction, suspected ischemia or necrosis, perforation, or acute abdomen, and its goals are to relieve obstruction, resect nonviable or clearly tumor-bearing bowel when necessary, and obtain adequate tissue without unnecessary delay to chemotherapy [4,6,9,13,16]. Recent pediatric series show that intussusception and abdominal mass are the main operative indications, that most children undergo segmental bowel resection with primary anastomosis, and that surgery remains crucial for establishing the diagnosis and enabling timely chemotherapy, although modern management discourages unnecessarily extensive resections because long-term outcome depends mainly on disease extent and rapid integration with multidisciplinary oncologic treatment [1,17,18,19].

## 2. Methods

This retrospective single-center case series included three children and adolescents who presented with acute intestinal obstruction or intussusception and were subsequently diagnosed with intestinal mature B-cell non-Hodgkin lymphoma (B-NHL). During the study period, a total of nine pediatric patients with B-cell non-Hodgkin lymphoma were treated at our institution, of whom only these three patients had intestinal involvement and presented with an acute surgical abdomen due to intestinal obstruction and/or intussusception and were included in the present case series. All patients were treated at the Department of Pediatric Surgery, University Hospital Centre Osijek, between July 2022 and July 2026.

Clinical data were retrospectively collected from medical records and included demographic characteristics, presenting symptoms and their duration, laboratory findings, imaging studies, preoperative diagnosis, indication for surgery, operative procedure and extent of bowel resection, postoperative course, histopathological findings, oncological treatment, follow-up, and clinical outcome.

All patients underwent abdominal imaging according to their clinical presentation. Urgent surgical intervention was performed because of acute bowel obstruction, intussusception, or suspected bowel ischemia. The surgical approach was individualized according to the intraoperative findings and aimed to relieve the acute complication, assess bowel viability, resect affected bowel when necessary, and obtain adequate tissue for definitive histopathological diagnosis. Following confirmation of B-NHL, all patients underwent further staging and were referred for multidisciplinary pediatric hematology–oncology management.

## 3. Case Presentation

### 3.1. Case 1—5-Year-Old, Jejuno-Jejunal Intussusception

**Figure 1:**
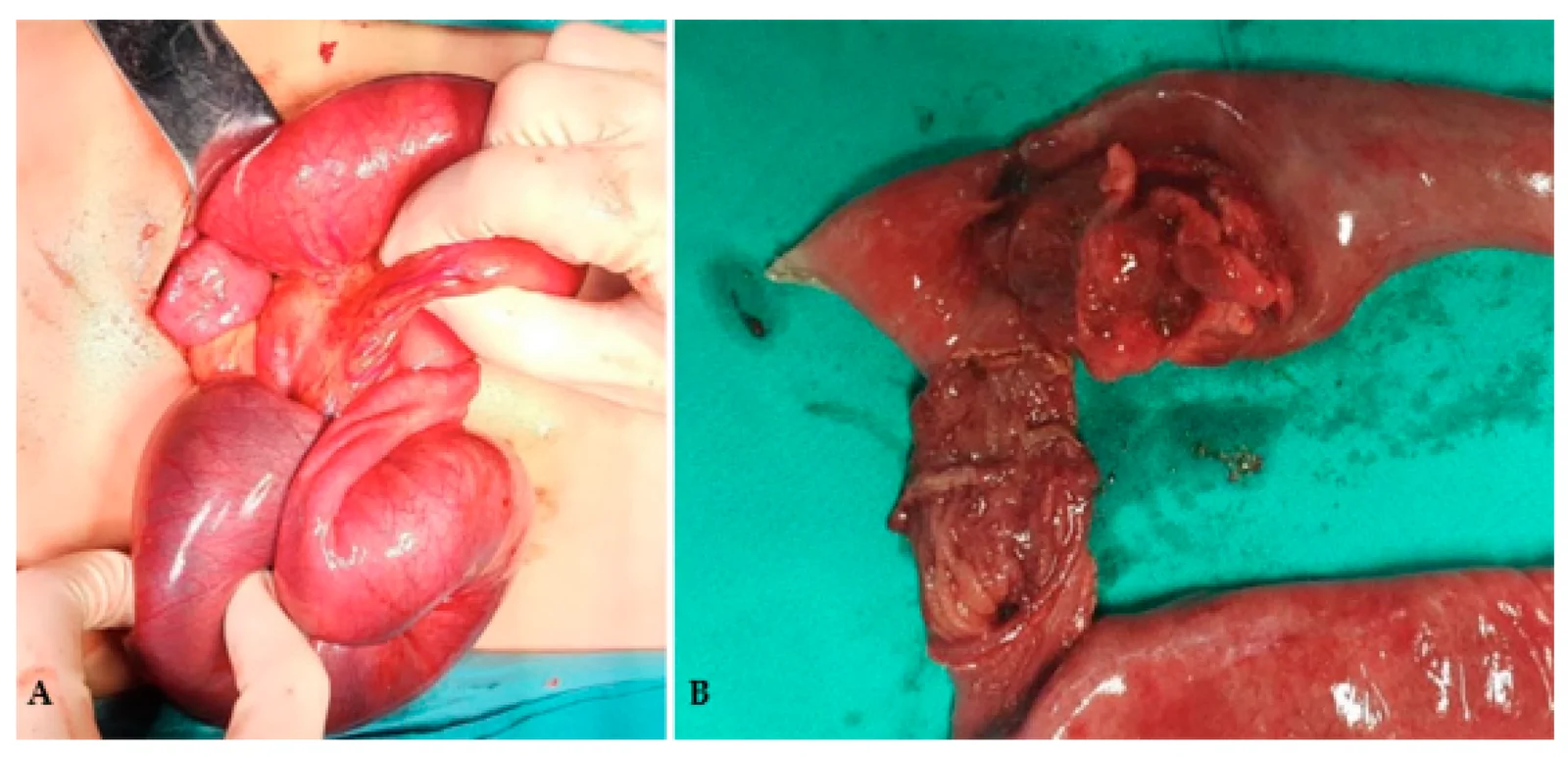
Intraoperative findings: (**A**) Jejuno-jejunal intussusception; (**B**) resected bowel segment with intraluminal mass serving as the lead point.

A previously healthy 5-year-old presented with sudden onset colicky abdominal pain, absence of bowel movement for two days and progressive distension. Clinically he appeared pale and exhausted, with palpable mass periumbilically. Laboratory findings were largely unremarkable, apart from an elevated lactate dehydrogenase (LDH) level of 454 U/L and leukocytosis with a white blood cell count of 21.3 × 10^9^/L. Two ultrasound examinations were performed. The initial ultrasound, performed by the on-duty emergency physician, raised suspicion of a jejuno-jejunal intussusception with a pathological lead point. The patient was subsequently referred to a specialist radiologist for a repeat examination. The second ultrasound demonstrated two ring-shaped bowel loops in the left upper abdomen, interpreted as being consistent with intussusception, although the exact bowel segment involved could not be reliably determined sonographically. A small amount of free fluid was noted in the pelvis and in the perisplenic region. No definitive sonographic signs of bowel necrosis were identified; however, bowel viability could not be reliably established by ultrasound. Given the clinical presentation with an acute abdomen, progressive abdominal distension and evolving intestinal obstruction, together with leukocytosis, free intraperitoneal fluid and concern for compromised bowel viability, urgent operative exploration was indicated. Moreover, the initial ultrasound suggested a jejuno-jejunal rather than a typical ileocolic intussusception, making pneumatic or hydrostatic enema reduction inappropriate. The suspected pathological lead point provided an additional indication for operative exploration. The nature of the suspected intraluminal lesion could not be established preoperatively and a neoplastic lesion could therefore not be excluded. Laparoscopy was chosen as the initial approach because the patient was clinically stable and it allowed direct confirmation of the diagnosis, assessment of bowel viability, and identification of the suspected pathological lead point through a minimally invasive approach. Laparoscopic exploration was initially performed; however, conversion to laparotomy was required because the intussusception could not be reduced laparoscopically. Manual reduction was successful and exposed a tumorous lesion approximately the size of an apricot as the lead point, with extensive ischemia and gangrene of the involved small-bowel segment, requiring segmental resection and primary anastomosis (Figure 1A,B).

Histology confirmed high-grade B-cell non-Hodgkin lymphoma demonstrating a characteristic “starry sky” pattern, with Ki-67 proliferation index 100%. Fluorescence in situ hybridization (FISH) analysis confirmed a MYC gene rearrangement at the 8q24 locus, establishing a definitive diagnosis of Burkitt lymphoma. Following diagnostic confirmation, the patient was urgently initiated on the mature B-NHL BFM protocol, beginning with the cytoreductive prephase (V-phase) alongside aggressive tumor lysis syndrome prophylaxis. Following completion of the NHL-BFM protocol, the patient achieved complete remission and remained disease-free during the subsequent three years of follow-up. The most recent MRI of the thorax, abdomen, and pelvis showed no evidence of active disease, and laboratory parameters were within normal limits.

### 3.2. Case 2—14-Year-Old with Obstructing Small-Bowel Mass

A previously healthy 14-year-old adolescent presented to the emergency department with a several-day history of progressively worsening crampy abdominal pain, obstipation, and vomiting on the day of arrival. On examination, the abdomen was mildly distended and tender in the lower abdomen, with high-pitched bowel sounds and no signs of peritonism; a suprapubic mass was palpable. Laboratory tests showed a white blood cell count of 11.4 × 10^9^/L, an LDH level of 180 U/L, and a C-reactive protein level of 65.2 mg/L, and appendicitis was initially suspected. A large, vascularized supravesical mass was identified on ultrasound, prompting further evaluation with CT. Pelvic CT revealed an intensely enhancing, 97 × 92 mm hypodense supravesical mass compressing adjacent small-bowel loops and resulting in secondary ileal distension. Associated findings included significant right-sided para-iliac, lobulated lymphadenopathy (up to 4.5 cm) and fat stranding; the appendix could not be visualized because of the mass effect. Based on the CT findings, a high suspicion of lymphoma was raised. At this stage, although the patient was clinically unwell, there were no findings requiring immediate emergency surgery, and acute appendicitis was excluded. The patient was therefore admitted for further pediatric evaluation and diagnostic workup of the suspected lymphoma. On the following day, before this workup could be completed, the patient’s clinical condition deteriorated significantly, with copious vomiting, increasing abdominal pain, progressive abdominal distension, and absence of bowel movements. The clinical and radiological findings were consistent with progression to mechanical intestinal obstruction and an acute abdomen, which constituted an indication for urgent surgical intervention. Although tissue biopsy for histopathological confirmation of the suspected lymphoma would otherwise have been considered as part of the diagnostic workup, the rapid clinical deterioration and development of symptomatic mechanical bowel obstruction precluded delaying treatment for a preoperative biopsy. Surgical exploration therefore served both therapeutic and diagnostic purposes, allowing relief of the obstruction and acquisition of tissue for definitive histopathological diagnosis. Emergency laparotomy was performed approximately 48 h after the patient’s initial presentation to the emergency department. Exploratory laparotomy demonstrated an intramural tumor mass forming a dense conglomerate of the small bowel and ascending colon, causing mechanical obstruction with significant dilation and vascular congestion of the proximal loops, without evidence of intestinal perforation. En bloc resection with primary anastomosis was performed, achieving complete macroscopic tumor excision. Postoperative recovery was uneventful. Immunohistochemical analysis demonstrated a Ki-67 proliferation index of 100%. Integrated morphological and immunophenotypic findings supported a diagnosis of diffuse large B-cell lymphoma (DLBCL, NOS) with a germinal center B-cell (GCB) phenotype. Fluorescence in situ hybridization (FISH) analysis showed no IGH/MYC rearrangement. The disease was associated with mesenteric, interaortocaval, para-aortic, pelvic, and cervical lymphadenopathy. Following BFM protocol treatment, complete remission was achieved, confirmed by a metabolically negative PET/CT. At the last follow-up, nearly four years following the initial diagnosis and surgical intervention, the patient remains in complete remission with no evidence of disease.

### 3.3. Case 3—17-Year-Old with Hematochezia

**Figure 2:**
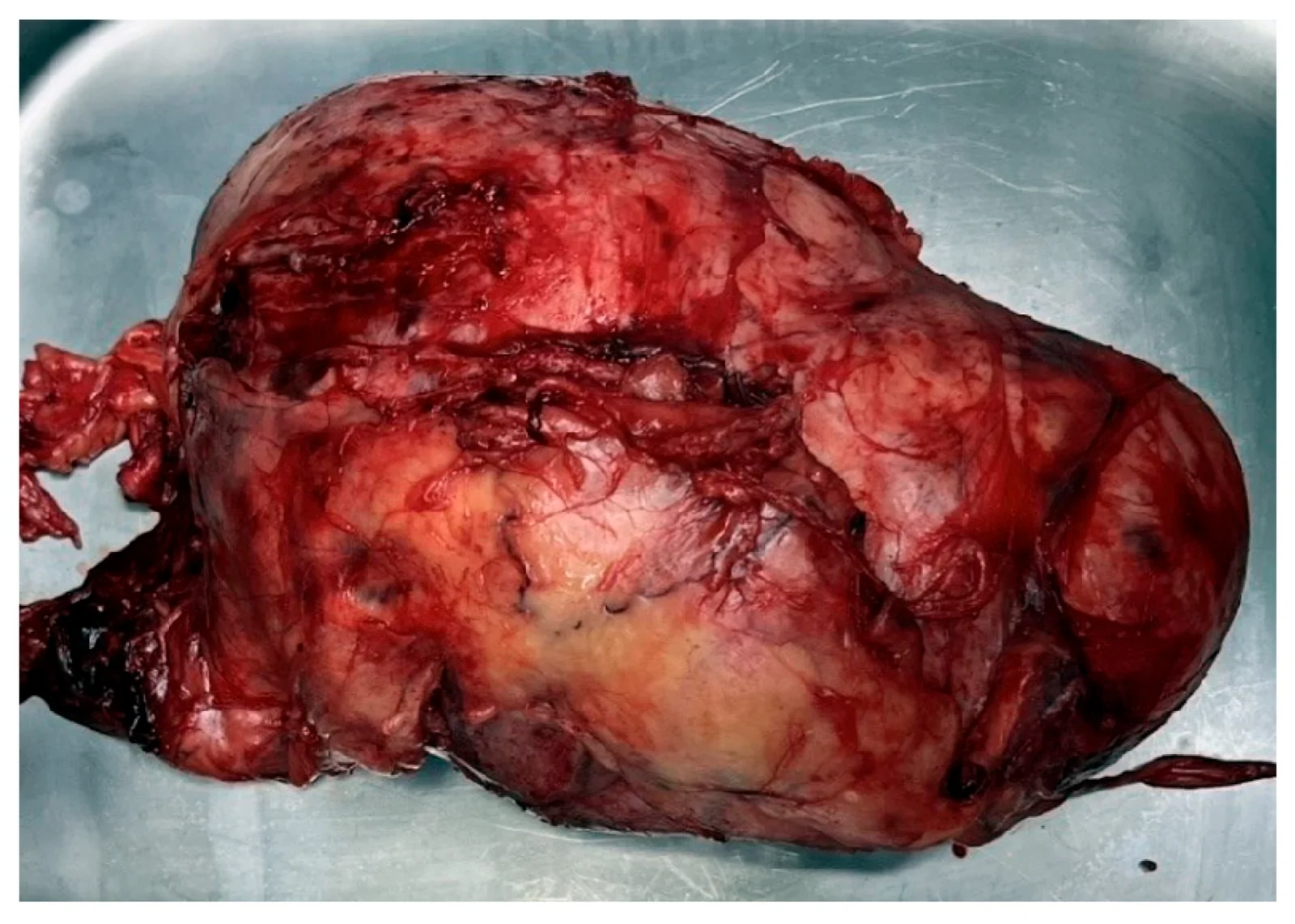
Large intraabdominal tumor mass causing bowel obstruction.

A 17-year-old obese male presented with a one-week history of cramping lower abdominal pain, non-bloody emesis, early satiety, an acute 8 kg weight loss, and hematochezia. Outpatient gastroenterology workup was pending. His medical history was notable for intermittent tachycardia managed with bisoprolol. Physical examination revealed lower abdominal tenderness, though body habitus limited the evaluation for palpable masses. Initial laboratory findings demonstrated a hemoglobin level of 119 g/dL, a C-reactive protein level of 12.4 mg/L, an LDH level of 230 U/L, and renal dysfunction secondary to dehydration, with a creatinine level of 122 µmol/L. Emergency colonoscopy revealed a large, hemorrhagic polypoid mass partially obstructing the ileocecal valve region, while abdominal and pelvic CT demonstrated a 7.5 cm, well-circumscribed solid mass interpreted as an appendiceal tumor. The radiological and endoscopic findings raised suspicion of an appendiceal neuroendocrine tumor associated with high-grade mechanical small-bowel obstruction and significant regional lymphadenopathy, requiring urgent surgical consultation. Given worsening pain and consistent nasogastric output, urgent surgery was undertaken 48 h after presentation. Urgent exploratory laparotomy identified a giant, solid mass involving the right colon and small bowel with no clear macroscopic origin, which caused multifocal luminal narrowing and kinking of the intestines with significant, extensive mesenteric lymphadenopathy (Figure 2).

At surgery, decompression of the obstructed small-bowel contents was performed. The indication for surgery was the high-grade mechanical bowel obstruction with progressive clinical symptoms in the setting of a large ileocecal/right-sided abdominal tumor that was preoperatively considered most likely to represent an appendiceal neoplasm. There was no bowel perforation or macroscopic evidence of bowel ischemia. Intraoperatively, however, the tumor could not be separated from the adjacent bowel loops, as it formed a firm conglomerate incorporating the right colon and adjacent small bowel and was responsible for multifocal luminal narrowing and kinking. Consequently, limited local excision of the mass was not considered technically feasible without compromising the involved bowel. A right hemicolectomy was therefore performed to relieve the obstruction and achieve complete removal of the macroscopically involved bowel and tumor. The enlarged regional lymph nodes were intimately adherent to and incorporated within the tumor–bowel conglomerate and were consequently removed en bloc with the specimen; no separate or extended lymphadenectomy was performed. Thus, the extent of surgery was dictated primarily by the anatomical involvement of the bowel and the need to relieve mechanical obstruction rather than by an attempt to perform an extended oncological resection for lymphoma. A primary anastomosis was subsequently performed. The postoperative recovery was uneventful; physiological peristalsis returned on postoperative day four, and the patient was subsequently discharged without complications. Histopathological analysis revealed a diffuse proliferation of atypical, medium-sized lymphocytes presenting a characteristic “starry sky” pattern. The tumor presented a 100% Ki-67 proliferation index and 100% c-MYC positivity, strongly favoring a high-grade mature B-cell lymphoma. Subsequent fluorescence in situ hybridization (FISH) analysis confirmed *MYC* gene rearrangement, confirming the definitive diagnosis of Burkitt lymphoma. This case highlights a diagnostic pitfall, as the radiological and endoscopic findings suggested an appendiceal neoplasm, whereas the definitive diagnosis was Burkitt lymphoma. Following histopathological confirmation of Burkitt lymphoma, the patient received systemic chemotherapy according to the GMALL B-ALL/NHL 2002 protocol, achieving complete remission. Subsequent outpatient hematology monitoring and a follow-up PET/CT scan demonstrated complete metabolic remission, with no evidence of residual disease or disease relapse, 50 months after the diagnosis.

The demographic, clinical, diagnostic, therapeutic, and outcome characteristics of all three patients are summarized in Table 1.

## 4. Discussion

These three cases demonstrate that pediatric intestinal mature B-cell non-Hodgkin lymphoma may first present as an acute surgical emergency rather than a recognized oncologic diagnosis. Although this pattern is uncommon, it is well recognized in the literature: intestinal obstruction or perforation occurred in 7.5% of children with intestinal mature B-cell lymphoma in one large pediatric series, while broader emergency literature confirms that intestinal lymphoma can present with obstruction, perforation, or other abdominal crises [8,20,21]. The clinical spectrum seen in these cases, from intussusception with ischemic bowel, to localized ileocecal obstruction, to a bulky mass causing mechanical bowel obstruction, reflects the heterogeneity of gastrointestinal Burkitt lymphoma presentations described across pediatric reports [1,7,22]. This is also biologically and clinically credible because the abdomen is the predominant primary site in many pediatric Burkitt lymphoma cohorts, reported in 56%, 62.5%, 73.9%, and 84.8% of cases across different series [23,24,25,26]. A central theme across these cases is the diagnostic obscurity of presentation since pediatric intestinal lymphoma often mimics far more common surgical or inflammatory conditions, and atypical abdominal presentations in older children can resemble idiopathic intussusception, appendicitis, nonspecific obstruction, or possible Meckel-related pathology [22]. Preoperative diagnosis remains difficult, and several reports note that definitive diagnosis is frequently established only after surgery and histopathologic examination of the resected specimen or biopsy material [4,7,9]. The present series also supports two recurrent emergency patterns. The first is pathologic lead-point intussusception, which should raise suspicion particularly in children older than four years, in recurrent cases, or when imaging suggests a lead point rather than idiopathic disease [11]. The second is tumor-related mechanical obstruction, in which direct bowel involvement or mass effect causes an acute abdomen without an obvious preoperative oncologic diagnosis [4,27]. From a surgical perspective, the literature supports a goal-directed emergency approach rather than routine extensive resection. In contemporary pediatric and mixed emergency series, surgery is primarily used to relieve obstruction, manage perforation or ischemia, obtain adequate tissue, and enable timely chemotherapy rather than to pursue radical extirpation as a stand-alone objective [1,4,16,27]. This principle is especially relevant in mature B-cell lymphoma, where delaying effective systemic therapy can be harmful and where modern treatment pathways depend on prompt histologic confirmation followed by coordinated oncologic care [4,25,28]. Our finding that bowel resection with primary anastomosis was feasible in all three patients describes the surgical management in this small series and should not be interpreted as evidence of an optimal operative strategy or of a specific prognostic effect of emergency surgery. Published studies have reported favorable outcomes in selected patients undergoing surgery for intestinal lymphoma, but these findings cannot be directly extrapolated to the present heterogeneous case series [1,17].

Emergency surgical management of intestinal B-cell lymphoma in children requires an individualized approach based on the clinical presentation, the extent of bowel involvement, and the patient’s overall condition. Although surgery is often necessary in the presence of complete obstruction, intussusception with a pathological lead point, bowel ischemia, or perforation, the operative strategy should be directed toward resolving the acute complication and obtaining adequate tissue for diagnosis rather than routine radical tumor resection. Intraoperative findings, including bowel viability, the extent of tumor involvement, and the possibility of achieving a safe reconstruction, should guide the choice between limited segmental resection, more extensive resection, or, in selected cases, biopsy or other limited interventions when immediate resection is not required. Primary anastomosis may be feasible when local and systemic conditions are favorable, whereas the need for diversion or delayed reconstruction should be considered in patients with severe contamination, compromised bowel viability, or unfavorable physiological conditions. Following surgery, rapid histopathological and molecular confirmation is essential to facilitate timely initiation of systemic chemotherapy. The optimal surgical approach should therefore be considered within a multidisciplinary framework, balancing the need for immediate control of the surgical emergency against the importance of avoiding unnecessary bowel loss and delays in definitive oncologic treatment. The main principles guiding emergency surgical decision-making, the extent of resection, and the transition to postoperative chemotherapy are summarized in Table 2.

Broader emergency data also suggest that, when intraoperative conditions are suitable, small-bowel resection with primary anastomosis offers acceptable short-term outcomes compared with other emergency procedures [20]. At the same time, the available evidence does not support unnecessarily aggressive upfront resection in all patients. Reviews of pediatric lymphoma surgery emphasize that extensive surgery can delay systemic treatment and may not achieve meaningful oncologic benefit when disease is disseminated or margins are unlikely to be clear [16]. Although some non-pediatric or mixed small-bowel lymphoma series report improved progression-free survival after gross or R0 resection, these findings derive from heterogeneous populations and do not outweigh the pediatric consensus that chemotherapy remains the main curative modality [4,29].

The broader oncologic literature further supports a restrained interpretation of these cases. Prognosis in pediatric mature B-cell lymphoma appears to depend more strongly on stage, tumor burden, CNS involvement, treatment delay, and supportive care capacity than on the emergency presentation itself [23,25,26].

Consistent with this, pediatric series have found that obstruction or perforation does not independently worsen survival, and even perforation was not associated with significantly inferior long-term outcomes when patients proceeded to definitive therapy [8,17,27]. This point is increasingly important because outcomes for pediatric mature B-cell lymphoma have improved substantially with risk-adapted multidisciplinary treatment. Contemporary reports describe survival approaching 90% in some settings, including a 5-year overall survival near 90% in developed-country experience and a 4-year overall survival of 92% in a modern LMIC cohort treated with reduced-intensity chemotherapy plus rituximab [4,28]. Recent series also incorporate PET/CT-based response assessment into treatment evaluation, underscoring that emergency surgery should be understood as one component of a larger diagnostic and therapeutic pathway rather than as definitive treatment by itself [4,28].

Intussusception in older children should prompt consideration of a pathological lead point. Similarly, an ileocecal mass associated with prominent regional lymphadenopathy should raise suspicion for lymphoma, even when radiological or endoscopic findings initially suggest another primary neoplasm. Emergency surgery should be directed toward resolving obstruction, treating ischemia/perforation and obtaining adequate tissue rather than pursuing unnecessarily radical resection. A minimally invasive approach may be attempted when technically feasible and clinically appropriate; however, if adequate exploration, safe relief of the obstruction, or appropriate tissue sampling cannot be achieved laparoscopically because of the intraoperative findings, conversion to an open approach should be undertaken without hesitation [30]. Rapid histopathologic and molecular diagnosis is essential to minimize delay in systemic therapy. Multidisciplinary surgical and oncologic management is central to achieving favorable outcomes.

This study has several limitations. The series comprises only three patients from a single institution, which limits generalizability and excludes statistical analysis. Second, the retrospective design is subject to incomplete documentation and selection bias. Third, the heterogeneity of clinical presentation, lymphoma subtype, disease stage, and surgical procedures limits direct comparison between cases. Lastly, the absence of a contemporaneous comparison group prevents assessment of whether emergency surgery independently influences oncologic outcomes. In addition, the present case series cannot determine the optimal surgical approach or establish the prognostic effect of emergency surgery. The literature review was narrative rather than systematic and therefore should not be interpreted as a formal synthesis of available evidence.

## 5. Conclusions

These cases demonstrate a rare but clinically important presentation of pediatric intestinal mature B-cell non-Hodgkin lymphoma, in which the initial manifestation is an acute surgical abdomen secondary to intussusception or bowel obstruction. They highlight the importance of including lymphoma in the differential diagnosis of older children and adolescents who present with atypical intussusception, unexplained mechanical obstruction, or an intra-abdominal mass in the emergency setting. In this context, surgery is best understood as an essential but primarily adjunctive intervention, serving to relieve acute complications, obtain diagnostic tissue, and facilitate transition to definitive oncologic management. Given the rarity of this presentation, further multicenter collaborative studies are warranted to better define its clinical spectrum, refine operative decision-making, and clarify how emergency surgical care is best integrated into contemporary pediatric lymphoma treatment strategies.

## Figures and Tables

**Table 1:** Summary of clinical cases: demographic, clinical and diagnostic characteristics.

Age/Sex	Presentation	Initial Impression	Imaging	Site	Surgery	Pathology	Therapy	Outcome
5/M	Colicky pain, obstipation, distension, palpable mass	Jejuno-jejunal intussusception	US: intussusception with suspected lead point	Jejunum	Laparoscopy converted to laparotomy, manual reduction, segmental resection, end-to-end anastomosis	Burkitt lymphoma; starry sky; Ki-67 100%; MYC+ FISH	B-NHL BFM Time to chemotherapy: 10 days	CR (36-month follow-up)
14/M	Crampy pain, obstipation, vomiting, suprapubic mass	Suspected appendicitis/obstructing mass	US/CT: supravesical mass, bowel compression, lymphadenopathy	Small bowel/ascending colon	Emergency laparotomy, en bloc resection, end-to-end anastomosis	DLBCL, NOS, GCB phenotype; Ki-67 100%; IGH/MYC FISH negative	BFM Time to chemotherapy: 9 days	CR (48-month follow-up)
17/M	Cramping pain, emesis, early satiety, weight loss, hematochezia	Suspected appendiceal NET	Colonoscopy/CT: ileocecal mass, appendiceal tumor, lymphadenopathy	Ileocecal region/appendix	Exploratory laparotomy, en bloc right hemicolectomy with incorporated regional lymph nodes, end-to-end anastomosis	Burkitt lymphoma; starry sky; Ki-67 100%; c-MYC 100%; MYC+ FISH	GMALL B-ALL/NHL 2002 Time to chemotherapy: 12 days	CR (50-month follow-up)

NET—neuroendocrine tumor; US—ultrasound; CT—computed tomography; B-NHL—B-cell non-Hodgkin lymphoma; DLBCL—diffuse large B-cell lymphoma; NOS—not otherwise specified; GCB—germinal center B-cell; FISH—fluorescence in situ hybridization; MYC—MYC proto-oncogene; IGH—immunoglobulin heavy chain; BFM—Berlin–Frankfurt–Münster protocol; GMALL B-ALL/NHL 2002—German Multicenter Study Group for Adult Acute Lymphoblastic Leukemia protocol; CR—complete remission. Time to chemotherapy refers to the interval between surgery and initiation of systemic chemotherapy, expressed in days.

**Table 2:** General considerations for emergency surgical decision-making in pediatric intestinal B-cell non-Hodgkin lymphoma.

Clinical Scenario	Possible Intraoperative Findings	Surgical Options	Rationale
Intussusception with a suspected pathological lead point	Tumor serving as lead point, bowel ischemia, necrosis	Reduction and segmental resection; primary anastomosis when feasible	Remove nonviable bowel, relieve obstruction, obtain diagnostic tissue
Complete mechanical bowel obstruction	Obstructing intraluminal or intramural mass, proximal bowel dilatation, viable or compromised bowel	Segmental resection with primary anastomosis; limited intervention or biopsy in selected cases	Relieve obstruction while avoiding unnecessary extensive resection
Perforation or peritonitis	Perforated tumor, contamination, inflamed or nonviable bowel	Resection of affected segment; anastomosis or diversion depending on conditions	Control contamination and remove nonviable bowel
Extensive or multifocal bowel involvement	Diffuse tumor infiltration, multiple involved segments, difficult reconstruction	Limited resection of symptomatic/complicated segment; biopsy when immediate resection is not required	Avoid excessive bowel loss and preserve options for systemic therapy
Stable patient with a suspicious mass but no acute complication requiring surgery	Localized or disseminated tumor, potentially resectable or unresectable disease	Biopsy or other diagnostic procedure rather than emergency resection	Establish diagnosis and avoid unnecessary surgery before chemotherapy
Postoperative period after diagnostic or therapeutic surgery	Histopathological and molecular confirmation of B-NHL	Prompt referral to pediatric hematology–oncology and initiation of risk-adapted chemotherapy	Minimize treatment delay and coordinate definitive oncologic care

B-NHL—B-cell non-Hodgkin lymphoma.

## Data Availability

The data assessed and reported here can be obtained from the authors upon reasonable request and following ethical and privacy principles.
